# Hypoxia reduces placental mTOR activation in a hypoxia‐induced model of intrauterine growth restriction (IUGR)

**DOI:** 10.14814/phy2.12651

**Published:** 2015-12-10

**Authors:** Rebecca Kimball, Montana Wayment, Daniel Merrill, Tyler Wahlquist, Paul R. Reynolds, Juan A. Arroyo

**Affiliations:** ^1^ Lung and Placenta Research Laboratory, Physiology and Developmental BiologyBrigham Young UniversityProvoUtah

**Keywords:** Hypoxia, IUGR, mTOR, placenta, trophoblast

## Abstract

Mammalian target of rapamycin (mTOR) is a protein that regulates cell growth in response to altered nutrient and growth factor availability. Our objective was to assess activated mTOR and its intracellular intermediates p70, and 4EBP1 in placental and invasive trophoblast cells in a hypoxia‐induced model of intrauterine growth restriction (IUGR) in rats. Rats were treated with hypoxia (9%) for 4 days. Placental and fetal weights, as well as conceptus numbers were recorded at the time of necropsy. Immunohistochemistry was used to determine the level of trophoblast invasion and apoptosis. Western blots were used to determine the activation of mTOR, p70, and 4EBP1 in the placenta and the uterine mesometrial compartment. We observed (1) decreased placental (21%) and fetal (24%) weights (*P* < 0.05); (2) decreased trophoblast invasion; (3) significantly increased active 4EBP1 (28%; *P* < 0.05) in invasive trophoblast cells yet no changes in the activation of mTOR and p70 proteins; and (4) a significant decrease in the activation of mTOR (48%; *P* < 0.05) with no differences in p70 or 4EBP1 activation in the placenta. We conclude that the development of IUGR is correlated with decreased activation of the mTOR protein in the placenta and increased 4EBP1 activity in the invading trophoblast. These results provide important insight into the physiological relevance of these pathways. Furthermore, modification of these and other related targets during gestation may alleviate IUGR severity.

## Introduction

Intrauterine growth restriction (IUGR) is an obstetric complication linked to an increased risk of morbidity and mortality for the fetus. This disease is characterized by low birth weight (below the 10th percentile) and it affects up to 10% of all pregnancies (Bahr et al. 2014). IUGR complications include perinatal hypoxia and asphyxia, neurological delays, and persistent pulmonary hypertension for the newborn (Brar and Rutherford 1988; Pollack and Divon 1992; Gray et al. 1999). This complication has also been linked to the adult onset of diabetes, hypertension, stroke, death from coronary vascular diseases, and can lead to preterm birth (Arroyo et al. 2008). Placental insufficiency is the most common cause of IUGR. Placentae in growth‐restricted pregnancies are pathologically characterized by reduced syncytiotrophoblast surface area, decreased trophoblast invasion, and increased placental trophoblast apoptosis (Krebs et al. 1996; Ishihara et al. 2002; Mayhew et al. 2003). Hypoxia has been thought to play an important role during IUGR. It has been shown that induced transient uteroplacental hypoxia causes significant IUGR suggesting a role localized hypoxia during IUGR (Tanaka et al. 1994). More recently, a study had shown that exposing rodents to hypoxia induced fetal weight reduction and IUGR, and induces metabolic and cardiovascular disturbances in adulthood, confirming an important role for hypoxia during this disease (Myatt 2006; Bourque et al. 2012; Giussani and Davidge 2013; Iqbal and Ciriello 2013; Rueda‐Clausen et al. 2014; Jang et al. 2015; Matheson et al. 2015). In vitro studies have demonstrated that hypoxia can affect the growth and differentiation of trophoblast cells, suggesting a role for hypoxia in trophoblast functioning and behavior (Genbacev et al. 1997; Nelson et al. 1999; Jiang et al. 2000; Caniggia and Winter 2002). To better study the molecular pathobiochemistry of IUGR, we chose to use a maternal hypoxia‐induced IUGR model in the rat. This model provides an essential tool to study trophoblast interactions in the uterine compartment, mTOR signaling, and apoptosis during hemochorial placentation. The rat uterine mesometrial compartment is the part of the uterus in which the blood vessel enters and models both endovascular and interstitial trophoblast invasion suggesting that, similar to humans, there is a specific function for trophoblast invasion during pregnancy in the rat (Soares et al. 2012).

The mammalian target of rapamycin (mTOR) protein regulates cell growth in response to the availability of nutrients and growth factors (Jansson et al. 2006; Wullschleger et al. 2006). Signaling intermediates of the mTOR pathway include the 70‐kDa ribosomal protein S6 kinase 1 (p70S6K) protein and the eukaryotic translation initiation factor 4E‐binding protein 1 (4EBP1) protein (Arroyo et al. 2009). Activation of these proteins regulates transcriptional control of target genes and protein synthesis necessary in the control of diverse response pathways (Arroyo et al. 2009). Studies have showed that the mTOR protein is increased during IUGR, whereas placental phospho (p)‐p70S6K protein is downregulated (Roos et al. 2007; Arroyo et al. 2009). Such differential responses suggest alternative mechanisms for these proteins in the placenta during IUGR that has not yet been adequately defined. Our laboratory has shown a direct correlation between mTOR protein activation and trophoblast invasion, suggesting a central role for this pathway in the regulation of trophoblast invasion during IUGR (Knuth et al. 2014). We hypothesized that maternal hypoxia will induce IUGR by regulating proteins associated with the mTOR pathway. In the present study, we characterized the family of signaling proteins regulated by mTOR in the placenta and in the uterine mesometrial compartment during a hypoxia‐induced IUGR.

## Material and Methods

### Animals and tissue preparation

A total of 14 weight‐matched (~400 g) Holtzman Sprague Dawley rats (HSD) were used for this study, which was approved by the Brigham Young University Animal Care and Use Committee (IACUC). To obtain timed pregnancies, females were caged with HSD males overnight. The presence of sperm in a vaginal smear was designated as day 0.5 of pregnancy. Placentae and uterine mesometrial compartments were dissected from pregnant rats at the time of necropsy (18.5 days of gestation; dGA). Dams, placentae, and fetuses were weighed (for each an average of 5 per litter) and tissues were snap frozen in liquid nitrogen for RNA and protein analysis. For immunohistochemistry (IHC) analysis, whole concepti were frozen in dry ice‐cooled heptane. All tissue samples were stored at −80°C until used.

### Environmental chamber and hypoxia exposure

To induce IUGR, pregnant rats were placed in a hypoxic chamber (*n* = 7) for 4 days (14.5–17.5 dGA). The treatment consisted of animal treated with hypoxic conditions of 9% O_2_. Exposed animals were labeled HX (*n* = 7; maternal weight 426 ± 12 g) and control (*n* = 7; maternal weight 393 ± 7 g) were maintained at 21% O_2_. Animals were euthanized (CO_2_ asphyxiation and cervical dislocation) at day 18 dGA when necropsies were performed.

### Immunohistochemistry

Immunohistochemistry (IHC) was performed on frozen whole conceptus sections as previously performed in our laboratory (Arroyo et al. 2009). In summary, slides were blocked with Sniper (Biocare Medical, Concord, CA) and incubated for 1 h with a rabbit polyclonal primary antibody against Cytokeratin 7 (CK7; Dako, Carpinteria, CA) for trophoblast localization, anti‐cleaved (active) caspase 3 (rabbit, Cell Signaling, Danvers, MA) antibody to assess apoptosis, anti‐phospho mTOR (Abcam, Cambridge, MA), anti‐phospho 4EBP1 (Abcam, Cambridge, MA), or with a universal IgG‐negative control (Biocare Medical; Concord, CA). Slides were incubated with Mach 2 universal stain polymer (Biocare Medical, Concord, CA) followed by color development with diaminobenzidine (DAB; brown color). Hematoxylin was used for nuclear counterstaining.

### RNA isolation

RNA was isolated using the Tri‐reagent method (Sigma, Saint Louis, MO) as suggested by the manufacturer. Briefly, placenta and uterine mesometrial compartment frozen tissues (100 mg) were homogenized in 1 mL of Tri‐reagent, and chloroform was added. The supernatant was transferred to a clean tube prior to the addition of cold isopropanol. Pellets were visualized and washed in 75% DEPC/EtOH solution and allowed to air dry for 10 min. Pellets were then resuspended in 50 *μ*L of DEPC water and RNA was quantified using a Nanodrop.

### Real time PCR

Real time PCR (RT‐PCR) was performed to determine the activation of the mTOR and mTOR signaling associated genes. cDNA was synthesized using Oligo (dT) and SuperScript II Reverse Transcriptase (both from Invitrogen by Life Technologies, Carlsbad, CA) by following the protocol suggested by the manufacturer. RT‐PCR was performed using SsoFast EvaGreen Supermix (Bio‐Rad Laboratories, Hercules, CA), which contains a cocktail of all necessary components excluding primers and templates. Sso7d‐fusion polymerase was used as the enzyme. Primers for rat mTOR (Fwd – ACCAATTATACTCGCTCCCTG, Rev – GTCATAGCAACCTCAAAGCA), p70 (Fwd – CAGAGCGGAATATTCTGGAG, Rev – CATAAATAGTTCTCCTCCACTGAG), and 4EBP1 (Fwd – GATGAGCCTCCCATGCAG, Rev – CCATCTCAAACTGTGACTCTTCA) were utilized with 18S primers (Fwd – GGGAGGTAGTGACGAAAAATAACAAT, Rev – CCCTCCAATGGATCCTCGTT) as a baseline control for the various experiments. Results (ΔΔ CT) were tested for significance against control animal tissues. Cycling conditions were as follows: 95°C for 30 sec; 95°C for 5 sec; 60°C for 30 sec; melt curve, 65°C for 2 sec and 95°C for 5 sec. An mTOR PCR array (PARN‐098Z; Qiagen, Valencia, CA) was performed to identify other genes related to mTOR signaling potentially affected by hypoxia.

### Western blot analysis

Western blot analysis was used to determine expression of the mTOR family of proteins in the placenta and uterine mesometrial compartment of control and treated animals as previously shown (Arroyo et al. 2009). Cell lysates (50 *μ*g) were separated on 4–12% Bis‐Tris gel SDS‐PAGE and transferred to nitrocellulose membranes. Membranes were incubated with antibodies against phospho mTOR (Ser2448), total mTOR, phospho p70 S6 kinase (SK6) (Thr389), total p70^SK6^, phospho 4EBP1 (Thr37/46), and total 4EBP1 (all from Cell Signaling Technology, Danvers, MA, excluding total p70 from Epitomics, Burlingame, CA). Membranes were then incubated with a secondary horseradish peroxidase (HRP)‐conjugated antibody for 1 h at room temperature. The membranes were incubated with ECL substrate, and the emission of light was detected using x‐ray film. To determine loading consistencies, each membrane was stripped and reprobed with an antibody against mouse *β* actin (Sigma Aldrich, St. Louis, MO). Expression levels of the proteins were quantified by densitometry normalized to *β* actin expression and changes in expression compared to the untreated controls were reported.

### Statistical analysis

Results were checked for normality and data are shown as means ± SE. Wilcoxon rank‐sums test was used to compare RNA and protein differences between groups, and *P *<* *0.05 was considered significant.

## Results

### Fetal and placental weights

**Figure 1:**
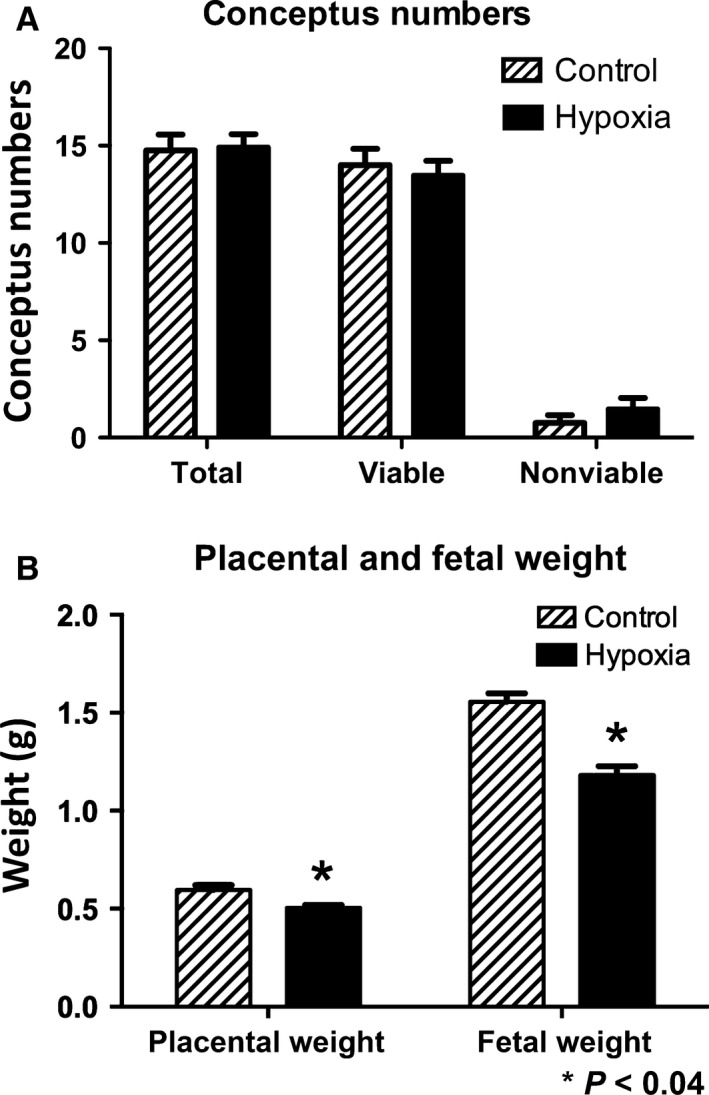
Placental and fetal weight differences during maternal hypoxia treatment in the rat. (A) A significant decrease in placental (1.2‐fold; *P* < 0.002) and fetal weights (1.3‐fold; *P* < 0.003) was observed in hypoxia (9% O_2_)‐treated animals as compared to controls (21% O_2_). (B) There were no significant differences in viable and nonviable fetuses between treated and control animals. *P ≤* *0.05.

Intrauterine growth restriction (IUGR) is characterized by decreased fetal and placental weight; therefore, we first investigated the effects of maternal hypoxia treatment on placental and fetal weights during pregnancy. Studies were performed exposing pregnant animals from a rage of 8–10% O_2_ conditions (data not shown). Exposing animals to 9% O_2_ was chosen as this was the lowest oxygen level treatment with no significant effects in viable concepti numbers as compared to controls (Fig. 1A). We found a 1.3‐fold reduction in fetal weight (*P* < 0.003) with a 1.2‐fold reduction in placental weight (*P* < 0.002) in rats exposed to hypoxia at the time of necropsy (Fig. 1B). These data supported a role for maternal hypoxia in fetal and placental weight deviations in this model of IUGR.

### Trophoblast invasion and apoptosis

**Figure 2:**
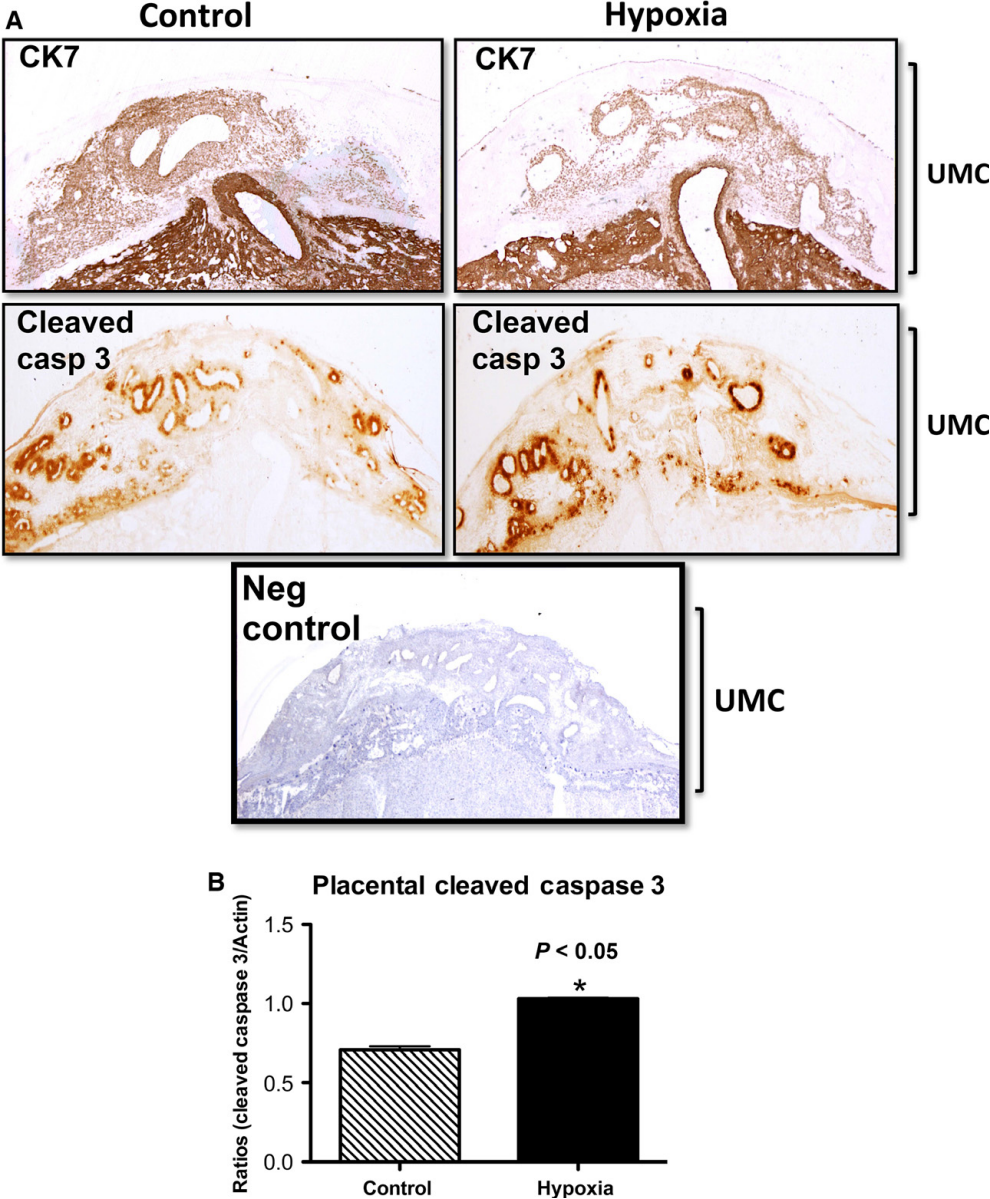
Trophoblast invasion and apoptosis during hypoxia treatment in the rat. (A) CK7 IHC showed decreased trophoblast invasion into the uterine mesometrial compartment (UMC) of treated animals as compared controls. Active caspase 3 IHC demonstrated increased apoptosis in invasive trophoblasts in the UMC of the treated animals when compared to controls. (B) Active caspase 3 was increased with maternal hypoxia in the placenta of treated animals as compared to controls. *P ≤* *0.05.

Shallow invasion of the trophoblast and increased placental apoptosis are hallmarks of IUGR. We accordingly investigated trophoblast invasion and apoptosis in the placenta during maternal‐induced IUGR. Cytokeratin 7 (CK7) was used to identify the localization of trophoblast cells in the placental villi. CK7 IHC showed decreased invasion of trophoblast cells into the uterine mesometrial compartment in hypoxia‐exposed animals compared to controls (Fig. 2A top panels). We next investigated whether apoptosis of the invading trophoblast cells was affected by hypoxia exposure. To achieve this, we immunostained for cleaved (active) caspase 3, a protein implicated in apoptosis. Hypoxia treatment showed an increased active caspase 3 staining in the invading trophoblast cells compared to controls (Fig. 2A bottom panels). Immunoblotting for active caspase 3 was performed in the placenta to semiquantitatively determine caspase 3‐mediated apoptosis. We observed a 1.5‐fold (*P* < 0.05) increase in placental active caspase 3 in treated animals when compared to controls (Fig. 2B). Our results suggested that hypoxia is likely involved in decreased trophoblast invasion and increased apoptosis observed in IUGR.

### mTOR family of proteins in the placenta and uterine mesometrial compartment

**Figure 3:**
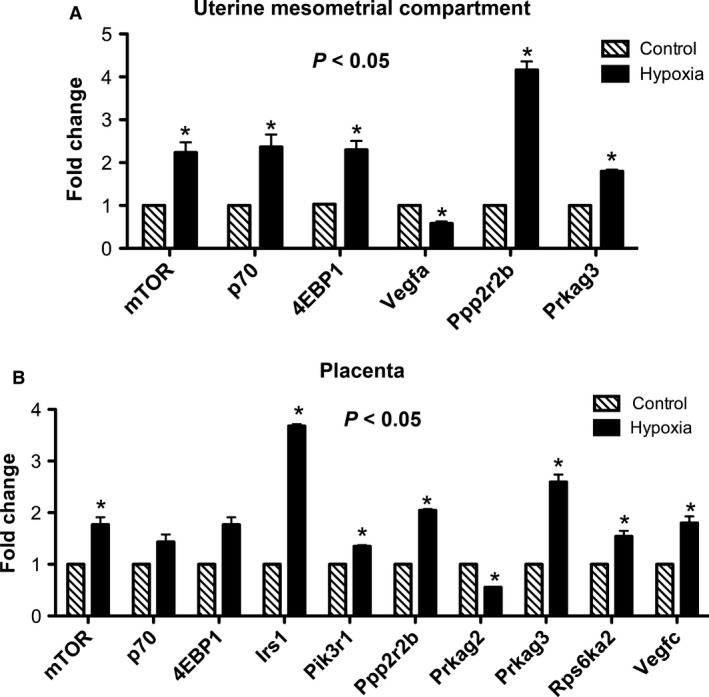
Uterine mesometrial compartment and placental mTOR gene activation during hypoxia in the rat. mTOR, p70, 4EBP1, Ppp2r2b, and Prkag3 genes were significantly induced (*P *<* *0.05) in the uterine mesometrial compartment of the hypoxia‐treated animals when compared to controls (A; *P ≤* *0.05). In the placenta, hypoxia induced a significant increase (*P *<* *0.05) in mTOR, Irs1, Pik3r1, Ppp2r2b, Prkag3, Rps6ka2, and VEGF‐C and a significant decrease (*P* < 0.05) in Prkag2 in the treated animals when compared to controls (B; *P ≤* *0.05).

**Figure 4:**
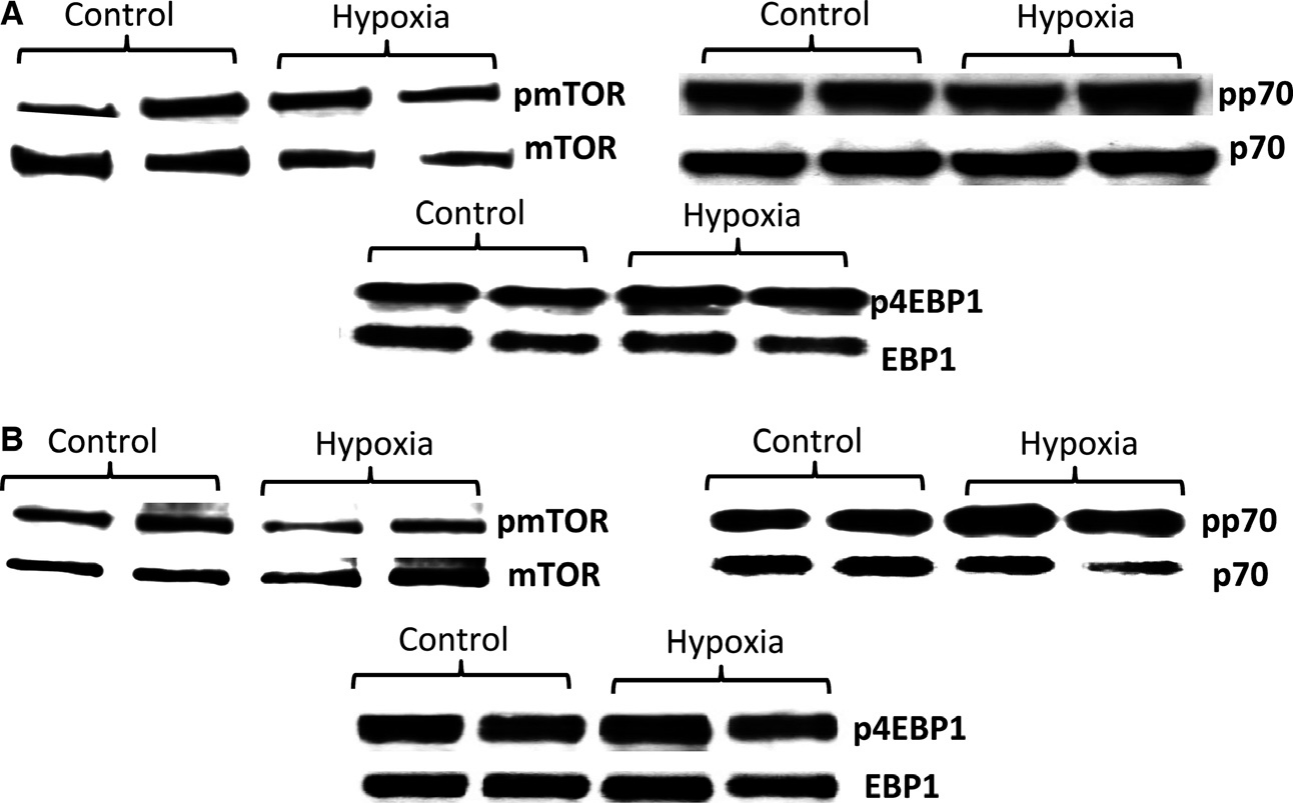
Characteristic western blot for mTOR, p70, and 4EBP1 proteins in the uterine mesometrial compartment (A) and placenta (B) of control and treated animals.

**Figure 5:**
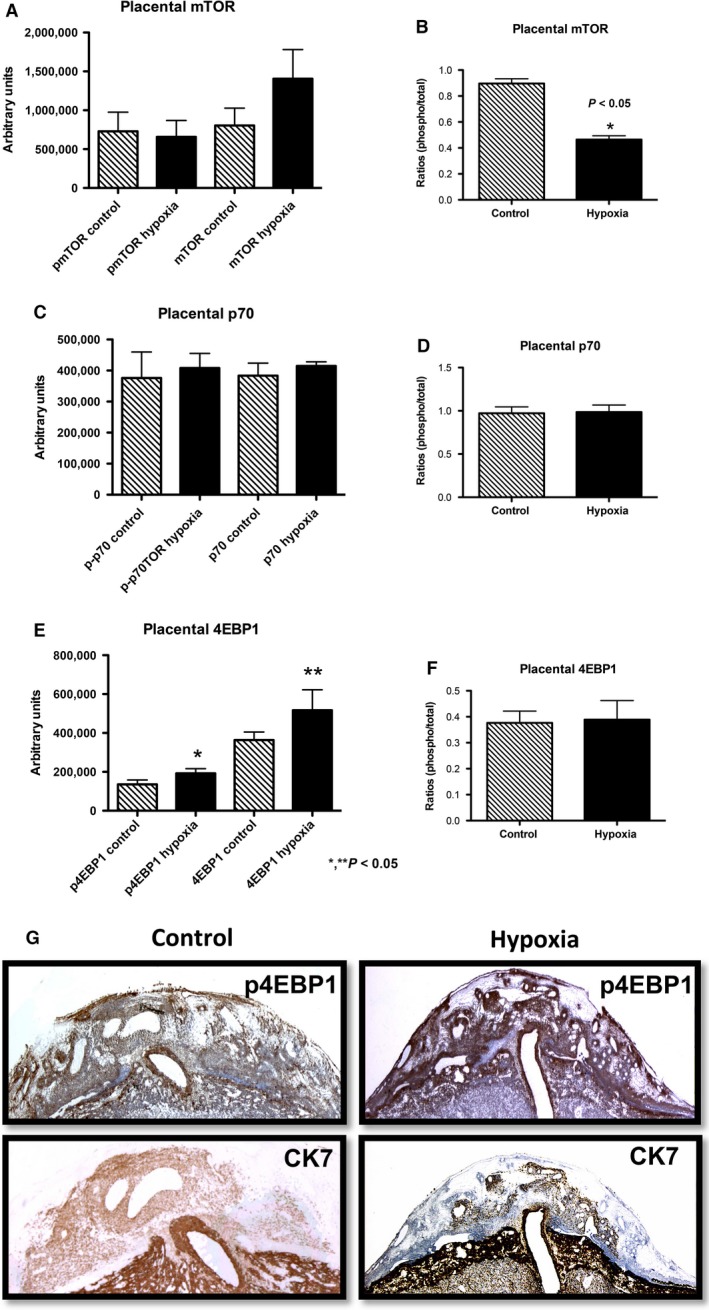
Activation of mTOR, p70, and 4EBP1 proteins in the uterine mesometrial compartment during hypoxia treatment in the rat. There were no differences in mTOR (A) or p70 (B) activation in the uterine mesometrial compartment during maternal hypoxia treatment. In contrast, there was a significant increase in 4EBP1 activation (1.8‐fold; *P* < 0.05) in the uterine mesometrial compartment of treated rats when compared to controls. (C) 4EBP1 activation was localized to the invasive trophoblast of the uterine mesometrial compartment (UCM) of the hypoxia‐treated animals. *P ≤* *0.05.

To precisely clarify mTOR gene expression patterns during hypoxia‐induced IUGR, we performed real time PCR using RNA isolated from the uterine mesometrial compartment. We observed a significant increase (2.2‐fold; *P* < 0.05) in the expression of active mTOR, p70, and 4EBP1 (Fig. 3A) in the hypoxia group when compared to controls. An evaluation of mTOR‐related genes led to the finding that vascular endothelial growth factor A (VEGF‐A) was decreased (1.7‐fold; *P* < 0.05) while significant increases were observed for the mRNA of the protein phosphatase, regulatory subunit B (Ppp2r2b; 4.0‐fold, *P* < 0.05) and the protein kinase, AMP‐activated, gamma 3 noncatalytic subunit (Prkag3; 1.8‐fold, *P* < 0.05) in the uterine mesometrial compartment of hypoxia‐exposed rats (Fig. 3A). We next investigated placental mTOR family gene expression. We observed increased mTOR mRNA (1.8%; *P* < 0.05) in exposed animals compared to controls (Fig. 3B). There were no significant changes in the expression of active p70 or 4EBP1 in exposed animals compared to controls (Fig. 3B). PCR array of the mTOR‐related genes revealed increased insulin receptor substrate 1 (Irs1; 3.7‐fold, *P* < 0.05), phosphoinositide‐3‐kinase, regulatory subunit 1 (alpha) (Pik3r1; 1.3‐fold, *P* < 0.05), protein phosphatase regulatory subunit B (Ppp2r2b; 2.0‐fold, *P* < 0.05), protein kinase, AMP‐activated, gamma 3 noncatalytic subunit (Prkag3; 2.6‐fold, *P* < 0.05), ribosomal protein S6 kinase polypeptide 2 (Rps6ka2; 1.5‐fold, *P* < 0.05), and the vascular endothelial growth factor C (VEGF‐C; 1.8‐fold, *P* < 0.05) genes (Fig. 3B). Only the protein kinase, AMP‐activated, gamma 2 noncatalytic subunit (Prkag2) gene was decreased (1.8‐fold, *P* < 0.05) in the placenta during maternal hypoxia treatment (Fig. 3B). To determine the protein changes in the mTOR‐related proteins western blot was preformed. Figure 4 shows a characteristic western blot for the phospho and total mTOR, p70, and 4EBP1 in the uterine mesometrial compartment (Fig. 4A) and the placenta (Fig. 4B) of treated animals as compared to control. We first investigated the protein levels of mTOR, p70, and 4EBP1 in the uterine mesometrial compartment. Immunoblotting demonstrated a significant increase in 4EBP1 activation (1.8‐fold; *P* < 0.05) in treated animals compared to controls (Fig. 5C). IHC was performed to confirm that 4EBP1 was spatially expressed in the invading trophoblast of the uterine mesometrial compartment. Phospho 4EBP1 IHC was increased (Fig. 5D, top panels) in the uterine mesometrial compartment of treated animals when compared to controls. This activation was present in invasive trophoblast cells (Fig. 5D, bottom panels). There were no significant differences in the expression of active mTOR or p70 between treated and control animals (Fig. 5A and B). No changes in the expression of these markers suggested plausible posttranscriptional regulation of mTOR and p70 in the uterine mesometrial compartment in hypoxia‐induced models of IUGR.

**Figure 6:**
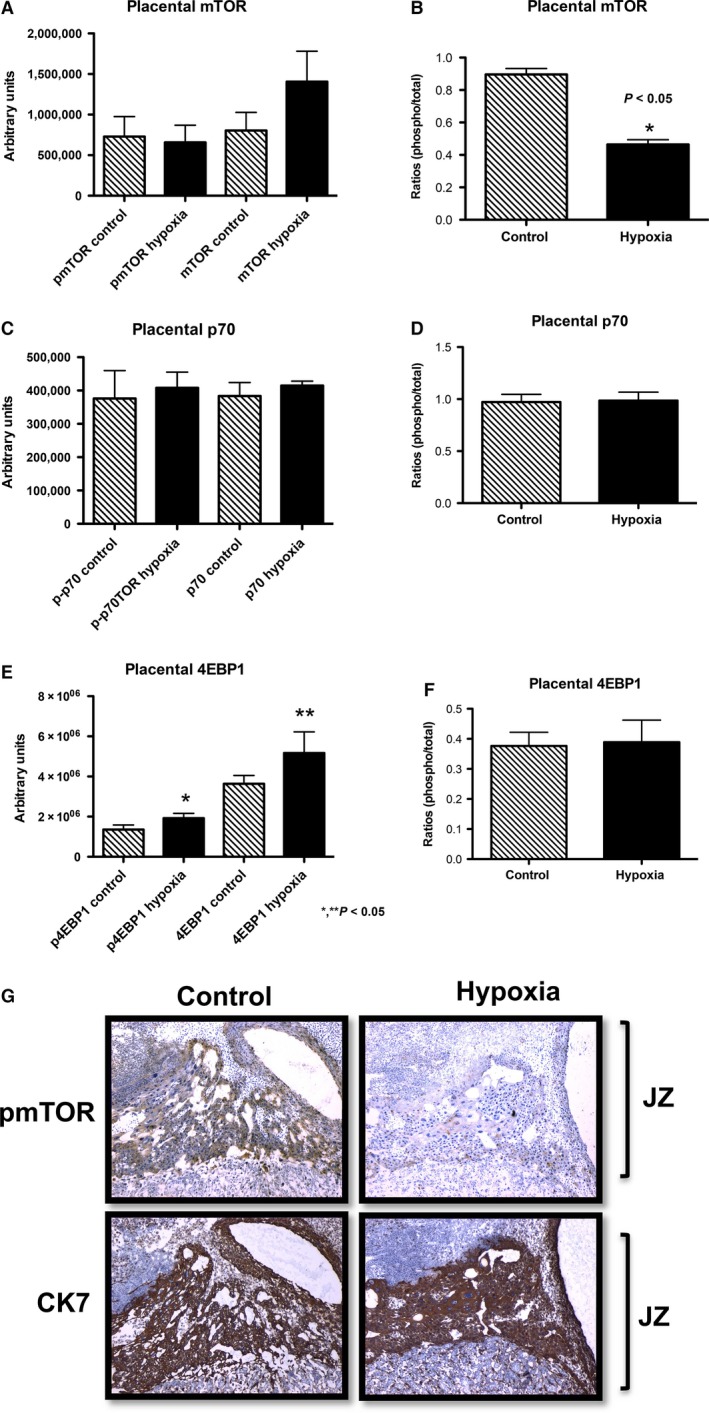
Activation of mTOR, p70, and 4EBP1 proteins in placenta during hypoxia treatment in the rat. There was a significant hypoxia‐induced decrease in mTOR protein activation (1.9‐fold; *P* < 0.05) (A). In contrast, there were no significant differences in p70 (B) or 4EBP1 (C) in the placenta of treated rats when compared to controls. Decreased mTOR activation was localized to the trophoblast in the junctional zone (JZ) of the placenta of the hypoxia‐treated animals when compared to controls. *P ≤* *0.05.

In the placenta, expression of active mTOR protein was significantly decreased (1.9‐fold; *P* < 0.05) in exposed animals compared to controls (Fig. 6A). However, there was no difference in the expression of p70 and 4EBP1 proteins (Fig. 6B and C) between the groups. Phospho mTOR IHC was performed in the placenta to determine if decreased active mTOR localized to trophoblast cells. Phospho mTOR staining showed that decreased mTOR protein expression was significantly localized to the junctional zone (JZ) of the placenta where robust endocrine cells are located. The JZ is also where invasive trophoblast cells are suggested to originate (Fig. 6D) (Ain et al. 2005). Ck7 IHC was performed to confirm the identity of the trophoblast cells present in the JZ. These compelling data suggested a function for placental mTOR activation in the development of IUGR during hypoxia.

## Discussion

Hypoxia is known to be associated with the development of IUGR (Tapanainen et al. 1994). We have chosen a hypoxia‐induced IUGR model in the rat in order to study molecular signaling pathways potentially involved in the progression of IUGR. In vivo maternal hypoxia treatment induced significantly decreased fetal and placental weight, decreased trophoblast invasion, and elevated apoptosis, all characteristics of IUGR. This treatment did not affect the conceptus number in the treated animals compared to the control animals. These observations support the fact that hypoxia treatment is a factor sufficient to induce IUGR characteristics in pregnant rats during hemochorial placentation. It is important to mention that previous publications have shown possible sex‐induced differences for hypoxia‐treated offspring (Giussani and Davidge 2013; Matheson et al. 2015; Reyes et al. 2015; Shah et al. 2015). A limitation of our study is the fact that fetal sex was not differentiated at the time of necropsy, but the decrease in fetal weights were obtained from fetuses obtained from the two uterine horns of each pregnancy.

Because IUGR pregnancies are characterized by decreased nutrients delivered to the fetus, we next investigated the mTOR family of proteins. The mTOR pathway of proteins is known to regulate cell growth in response to nutrients and growth factors (Blume‐Jensen and Hunter 2001; Jansson et al. 2006; Wullschleger et al. 2006). Previous research has identified plausible roles for mTOR in IUGR; however, no definitive studies have been performed that evaluate its family members in the low oxygen tension placenta. In the present study, we investigated the mTOR pathway in both the placenta and uterine mesometrial compartment in normal and hypoxia‐induced IUGR pregnancies. The uterine mesometrial compartment showed an increase in mTOR, p70, and 4EBP1 mRNA expression suggesting IUGR from hypoxia‐treated animals is mediated, at least in part, by the activation of the mTOR family of genes in the uterine mesometrial compartment. Interestingly, we also observed increased expression of the Ppp2r2b and Prkag3 genes. These are both involved in the negative regulation of mTOR (Qian et al. 2015). Ppp2r2b activation is known to be involved in the negative control of cell growth and division. It is also involved in the downregulation of mTOR activation (Qian et al. 2015). Prkag3 is a regulatory subunit of the AMP‐activated protein kinase (AMPK) and it plays a role in the regulation of energy metabolism in the cells (Milan et al. 2000). AMPK regulates ATP synthesis and is known to inhibit mTOR activation (Rehman et al. 2014). In contrast, we observed decreased expression of VEGF‐A with hypoxia treatment. VEGF‐A is involved in vasculogenesis and angiogenesis and can act as a potent vasodilator in stable vasculature (Eriksson et al. 2002). Our results convey the concept that although maternal hypoxia activates the mTOR family of proteins, it also activates negative regulators of the mTOR signaling pathway and inactivates VEGF‐A, a common positive regulator of mTOR (Trinh et al. 2009).

Interestingly, we only observed a significant increase in active 4EBP1 protein in the invading trophoblast and not increased mTOR or p70. Such a discrepancy in mRNA and protein suggests possible posttranscriptional regulation for mTOR and p70, as previously postulated by others (Lechuga et al. 2004; Mura et al. 2015). Such regulation may perhaps include the activation of the Ppp2r2b and Prkag3 genes. Although the mTOR mRNA was upregulated, decreased active protein expression could lead to the overall condition of a poorly developed and immature fetus. It is possible that mesometrial tissues attempt to compensate for hypoxia by activating 4EBP1 protein, but such a response fails to activate this anabolic pathway.

In the placenta, however, we observed a much different response. Only mTOR mRNA was increased in the placenta. We also observed an upregulation in the mTOR‐positive regulators Irs1, Pik3r1, Rps6ka2, and VEGF‐C while there was a downregulation in the mTOR‐negative regulator Prkg2. Consistent with the observation in the uterine mesometrial compartment, we observed an upregulation of the Prkag3 gene. Both Irs1 and Pik3r1 are involved in insulin cellular processes (Pollack and Divon 1992; Wullschleger et al. 2006). Irs1 participates in the transduction of signaling initiated at the activation of insulin receptors and is required for cellular metabolism (Copps and White 2012). Pik3r1 is involved in the insulin‐mediated increase in glucose uptake (Miled et al. 2007). IUGR is characterized with decreased amino acids and growth factors, which can affect placental metabolism. Therefore, the activation of Irs1 and Pik3r1 is in accordance with a variety of mechanisms related to fetal metabolic adaptations. For example, increasing peripheral insulin sensitivity for glucose utilization and decreasing insulin sensitivity for protein synthesis during IUGR are plausible maternal responses in an attempt to ensure fetal survival during these low nutrients conditions (Thorn et al. 2011). Rps6ka2 and VEGF‐C genes are involved in promoting cell growth motility and survival, suggesting that, similar to Irs1 and Pik3r1, induction could be due to a survival mechanism triggered by hypoxic stress (Milan et al. 2000; Roux et al. 2003). In agreement with this concept are data related to a decreased placental expression of Prkag2, an mTOR‐negative regulator that is involved with AMP signaling (Stapleton et al. 1996).

When we studied placental activation of mTOR, p70, and 4EBP1, only mTOR protein activation was decreased in treated animals, while there were no differences in the expression of p70 and 4EBP1. IHC demonstrated that decreased active mTOR was more localized to the trophoblasts in the JZ of the placenta, a structure suspected to primarily give rise to invasive trophoblast cells. The importance of the JZ is underscored by the previous observation that decreased trophoblast invasion is observed when mTOR activation is inhibited (Knuth et al. 2014). We therefore conclude that IUGR is correlated with increased 4EBP1 activity in the invading trophoblast cells of the uterine mesometrial compartment, decreased activation of the mTOR protein, and decreased trophoblast invasion in the placenta during maternal hypoxia‐induced IUGR.

Our results could provide needed insight into the physiological relevance of these pathways in the pathogenesis of IUGR and suggest possible targets that may assist in the alleviation of IUGR. As extensions of our findings in the low oxygen tension placenta, additional research on the mTOR pathway and the alteration of associated proteins may further elucidate mechanistic progression factors for IUGR and provide a more effective modality for the therapeutic treatment of growth‐restricted fetuses.

## Conflict of Interest

None declared.
